# A juvenile subfossil crocodylian from Anjohibe Cave, Northwestern Madagascar

**DOI:** 10.7717/peerj.2296

**Published:** 2016-09-15

**Authors:** Joshua C. Mathews, Karen E. Samonds

**Affiliations:** Department of Biological Sciences, Northern Illinois University, DeKalb, IL, United States

**Keywords:** Madagascar, Anjohibe Cave, *Voay robustus*, *Crocodylus niloticus*, Subfossil, Pleistocene, Holocene

## Abstract

Madagascar’s subfossil record preserves a diverse community of animals including elephant birds, pygmy hippopotamus, giant lemurs, turtles, crocodiles, bats, rodents, and carnivorans. These fossil accumulations give us a window into the island’s past from 80,000 years ago to a mere few hundred years ago, recording the extinction of some groups and the persistence of others. The crocodylian subfossil record is limited to two taxa, *Voay robustus* and *Crocodylus niloticus*, found at sites distributed throughout the island. *V. robustus* is extinct while *C. niloticus* is still found on the island today, but whether these two species overlapped temporally, or if *Voay* was driven to extinction by competing with *Crocodylus* remains unknown. While their size and presumed behavior was similar to each other, nearly nothing is known about the growth and development of *Voay*, as the overwhelming majority of fossil specimens represent mature adult individuals. Here we describe a nearly complete juvenile crocodylian specimen from Anjohibe Cave, northwestern Madagascar. The specimen is referred to *Crocodylus* based on the presence of caviconchal recesses on the medial wall of the maxillae, and to *C. niloticus* based on the presence of an oval shaped internal choana, lack of rostral ornamentation and a long narrow snout. However, as there are currently no described juvenile specimens of *Voay robustus*, it is important to recognize that some of the defining characteristics of that genus may have changed through ontogeny. Elements include a nearly complete skull and many postcranial elements (cervical, thoracic, sacral, and caudal vertebrae, pectoral elements, pelvic elements, forelimb and hindlimb elements, osteoderms). *Crocodylus niloticus* currently inhabits Madagascar but is locally extinct from this particular region; radiometric dating indicates an age of ∼460–310 years before present (BP). This specimen clearly represents a juvenile based on the extremely small size and open sutures/detached neural arches; total body length is estimated to be ∼1.1 m (modern adults of this species range from ∼4–6 m). This fossil represents the only juvenile subfossil crocodylian specimen reported from Madagascar.

## Introduction

The island country of Madagascar is considered a hot spot for biodiversity due to its high level of endemism (Myers et al., 2000). While the island is currently under intense study by scientists researching the extant fauna, many questions still remain about when and how this fauna arrived (e.g., Samonds et al., 2013). Madagascar has been isolated in the Indian Ocean for over 80 million years and most of the fauna is thought to have arrived on the island during this time (Storey et al., 1995; Ali & Huber, 2010; Samonds et al., 2012).

Crocodylomorpha has a long evolutionary history dating back to the Late Triassic, ∼230 mya (Irmis, Nesbitt & Sues, 2013; Zanno et al., 2015), and have had a largely global distribution for most of their evolutionary history. The earliest known crocodylomorph fossils on Madagascar date back to the early Jurassic with the teleosaurid *Steneosaurus baroni* Newton, 1893. However the Late Cretaceous Maevarano Formation in the Mahajanga Basin (Buckley et al., 2000; Buckley, 2001) preserves a very diverse assemblage of notosuchian, mahajangasuchid, and trematochampsid crocodylomorphs that includes *Simosuchus clarki* Buckley et al., 2000, *Mahajangasuchus insignis* Buckley & Brochu, 1999, *Araripesuchus tsangatsangana* Turner, 2002, and *Miadanasuchus oblita* (Buffetaut & Taquet, 1979), (Buckley et al., 2000; Buckley, 2001; Krause et al., 2006; Rasmusson-Simons & Buckley, 2009; Turner, 2006; Turner & Sertich, 2010). While this Cretaceous assemblage is unique in its diversity, these groups of crocodylomorphs became globally extinct following the end Cretaceous mass extinction event (O’Connor et al., 2010).

In contrast to this Cretaceous assemblage, the Cenozoic fossil record of Madagascar is relatively unknown largely due to the lack of terrestrial deposits. Because of this, little is known about crocodylians from this time period. Today, the only living Malagasy crocodylian is the Nile crocodile, *Crocodylus niloticus* Laurenti, 1768 (Brochu, 2007), which has also been reported from subfossil assemblages (e.g., Bickelmann & Klein, 2009). The only other subfossil crocodylian reported from Madagascar is *Voay robustus* Grandidier & Vaillant, 1872, an extinct form from the Late Pleistocene and Holocene (Brochu, 2007).

Many subfossil sites (subfossil referring to the relatively young age and incomplete mineralization of the remains) across the island have produced crocodylian remains (Brochu, 2007; Muldoon et al., 2012; Goodman & Jungers, 2014; Rosenberger et al., 2015). However, due to the extensive interest in the origins of the elephant birds, lemurs and other mammals, subfossil crocodylians have been largely overshadowed. The near lack of Cenozoic fossils, and the recognition of only two crocodylian taxa in the subfossil record, *Voay robustus* and *Crocodylus niloticus*, has led to questions regarding whether they ever coexisted (Brochu, 2007; Bickelmann & Klein, 2009); some have suggested the recent form (*Crocodylus*) outcompeted *Voay* (Burney et al., 1997). Owing to the fact that very little is known about either of these genera on the island, a better understanding of their paleobiology is needed to more rigorously test this hypothesis.

**Figure 1:**
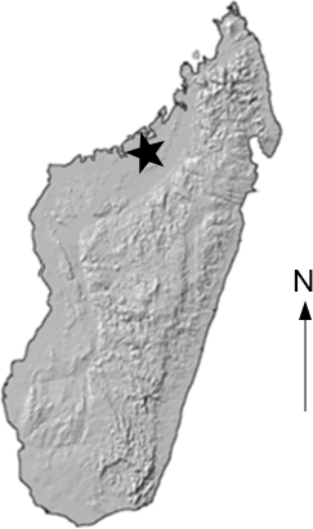
Locality Map. Map showing location of the Anjohibe Cave system in Northwest Madagascar.

**Figure 2:**
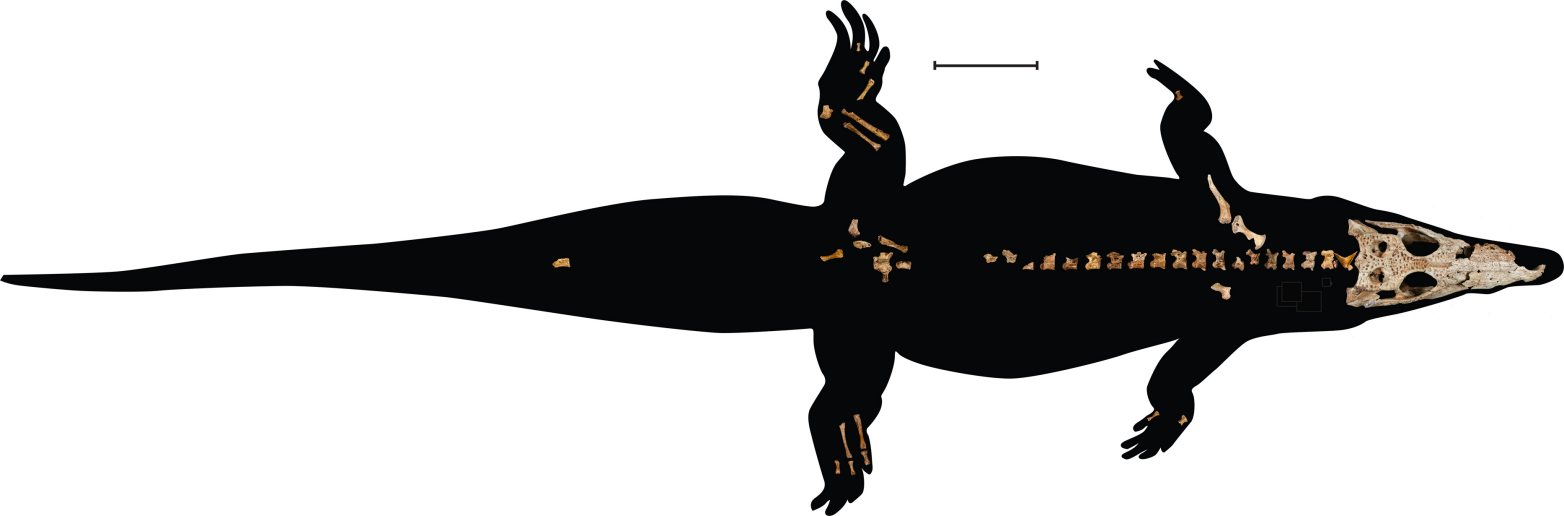
UAP-03.791, crocodylian specimen recovered from Anjohibe Cave, Northwestern Madagascar. Scale bar = 10 cm.

**Figure 3:**
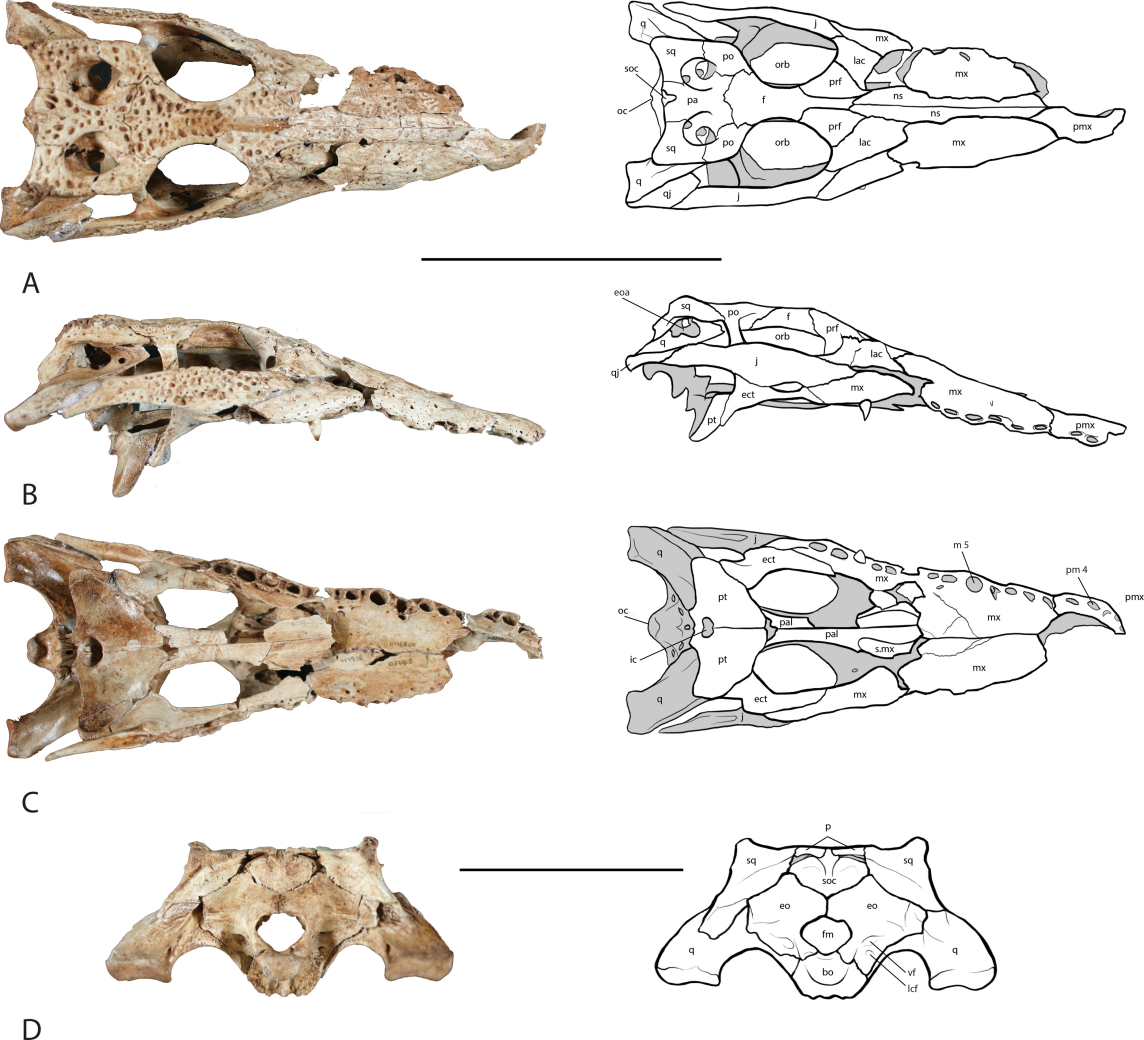
Skull of UAP-03.791. (A) dorsal view; (B) right lateral view;. (C) ventral view; (D) occipital view. Top scale bar = 10 cm; Bottom scale bar = 5 cm. Abbreviations: bo, basioccipital; ect, ectopterygoid; eo, exoccipital; eoa, external otic aperture; f, frontal; fm, foramen magnum; ic, internal choana; j, jugal; lac, lacrimal; lcf, lateral carotid foramen; mx, maxilla; m5, maxillary alveolus 5; ns, nasal; oc, occipital condyle; orb, orbit; pa, parietal; pmx, premaxilla; pm 4, premaxillary alveolus 4; po, postorbital; prf, prefrontal; pt, pterygoid; q, quadrate; qj, quadratojugal; s.mx, sutural surface on palatine for maxilla; soc, supraoccipital; sq, squamosal; vf, vagus foramen.

**Figure 4:**
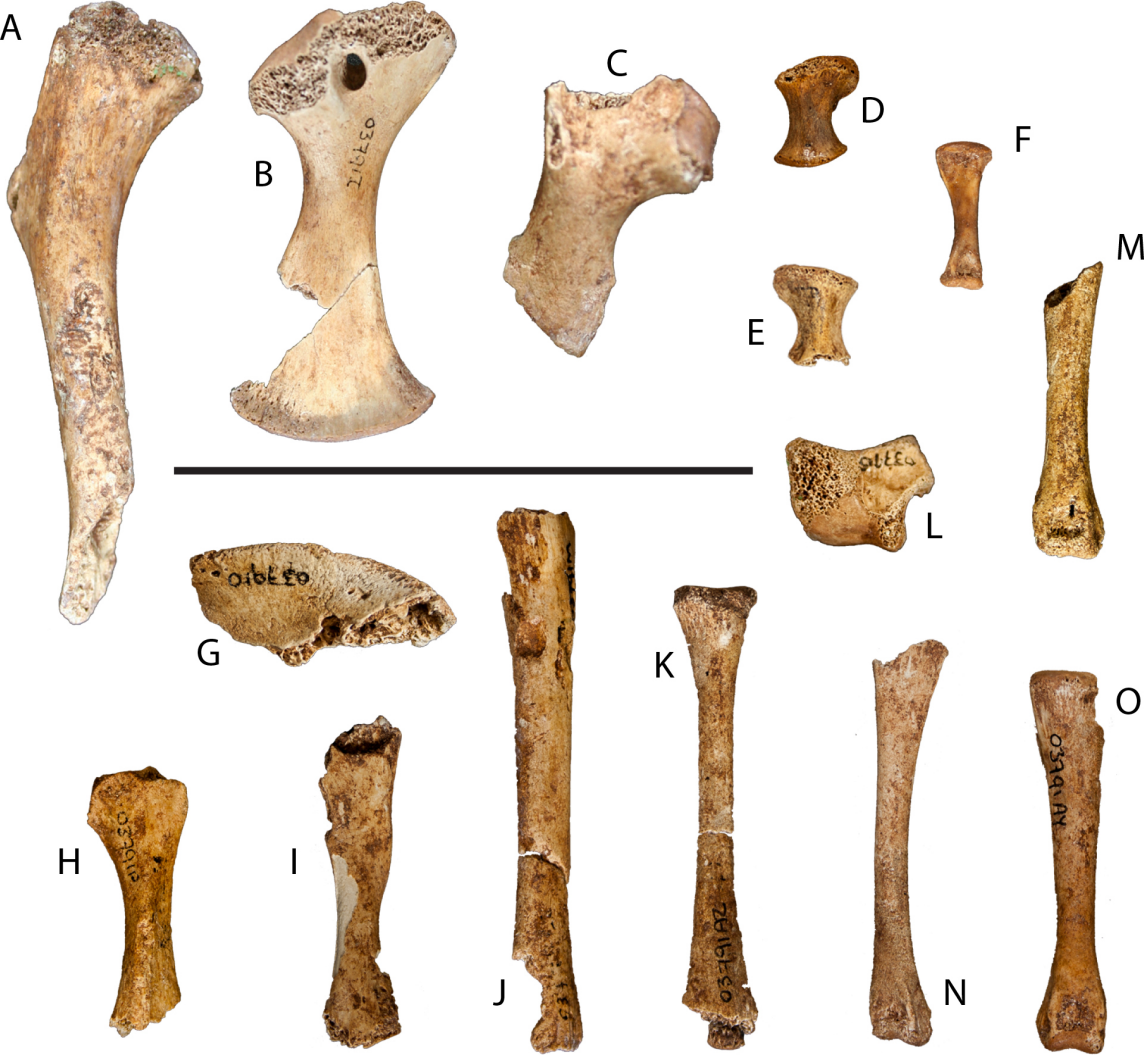
Postcranial remains of UAP-03.791. (A) Partial left humerus, dorsal view; (B) left coracoid, dorsal view; (C) partial right scapula, ventral view; (D) left radiale, dorsal view; (E) right radiale, dorsal view; (F) metacarpal, dorsal view; (G) partial left ilium, lateral view; (H) partial left ischium, medial view; (I) partial left pubis, medial view; (J) partial left tibia, dorsal view; (K) partial left fibula, dorsal view; (L) left calcaneum, lateral view; (M–O) left metatarsals. Scale = 5 cm

In 2003, an expedition to Anjohibe Cave in northwest Madagascar (Fig. 1) recovered the disarticulated remains of a small crocodylian from one small puddle within the cave system. The remains include a nearly complete skull and mandible, a nearly complete cervical series, most of the thoracic vertebrae, one sacral vertebra, one caudal vertebra, elements of the forelimb and hind limbs, elements of the pectoral and pelvic girdle, ribs, numerous osteoderms, and many isolated teeth (Figs. 2, 3 and 4) Radiometric dating has indicated that the specimen dates to ∼460–310 years BP (Crowley & Samonds, 2013).

The specimen described here, UAP-03.791, is a young individual due to the presence of open sutures and/or detached neural arches of the vertebral column. However, since all known specimens of *V. robustus* are from adult individuals, and we know virtually nothing of its growth and development, it is difficult to eliminate the possibility that the specimen could represent *Voay*. Both *C. niloticus* and *V. robustus* have been reported from Anjohibe Cave (Goodman & Jungers, 2014). While some postcranial elements of *Voay* differ from extant crocodylians (Brochu, 2007), these elements have not been recovered or are too incomplete to distinguish. In order to assign a proper diagnosis, we compare here the new juvenile specimen to those of young and adult *C. niloticus* and adult *V. robustus*, and use additional methods (e.g., CT scanning) to incorporate details of the growth and development of *Crocodylus* into our analysis.

## Geological Setting

Anjohibe Cave is located in northwestern Madagascar, northeast of Mahajanga (Fig. 1), and is found on the southern part of the current dry savannah of the Mahavo plains (Samonds, 2007). It is part of a large karst system that is formed within the Eocene limestone plateau with many kilometers of passages and over two dozen entrances (Burney et al., 1997; Samonds, 2007). Anjohibe has been extensively studied for decades and has provided us with some of the best evidence documenting environmental change in and around the cave over the last 40,000 years (Brook et al., 1999; Burney et al., 1997; Goodman & Jungers, 2014). Thousands of fossils have been collected from Anjohibe Cave; these include a diverse and very well preserved fauna including numerous species of birds, crocodiles, turtles, bats, rodents, tenrecs, hippos, carnivorans, the aardvark-like *Plesiorycteropus* and 11 species of lemur (e.g., MacPhee, 1994; Burney et al., 1997; Godfrey & Jungers, 2002; Samonds, 2007; Faure et al., 2010).

## Systematic Paleontology

**Table:** 

Eusuchia Huxley 1873
Crocodylia Gmelin 1789, sensu Benton & Clark 1988
Crocodylidae Cuvier, 1807
*Crocodylus niloticus* Laurenti, 1768

## Materials and Methods

Both dry and wet specimens of young *Crocodylus niloticus* were examined from collections at the University of California Museum of Paleontology, Berkeley, California and from the Amphibians & Reptiles collection at the Field Museum of Natural History, Chicago, Illinois. Both articulated and disarticulated skulls were examined so as to understand and identify disarticulated elements of the Malagasy specimen and how they might articulate with one another. Additionally, skulls from adult specimens of *C. niloticus* and *V. robustus* were examined from collections at the Université d’ Antananarivo Paléontologie in Antananarivo, Madagascar.

Because of the limited number of available skulls of young individuals, one wet specimen of *C. niloticus* was scanned using computed tomography (CT) to better visualize the internal anatomy. CT was performed with a Toshiba Aquilion 64 multislice CT scanner with 64 detector channels (Toshiba America Medical Systems, Inc., Tustin, CA) at Rockford Memorial Hospital, Rockford, Illinois, USA. Scanning thickness was prescribed at 0.50 mm with 0.25 mm overlapped image reconstruction. Image matrix was 512 × 512 × 16. The scanned volume data set was transferred to a computer workstation where volume rendered multiplanar and 3D images were created with Vitrea Software (Vital Images, Inc., Minnetonka, MN).

### Description

#### Skull

The right premaxilla is nearly complete preserving five alveoli (Fig. 3). It forms the anterior and lateral margin of the external naris and articulates posteromedially with a small anterior process of the nasal bones. A posterior process projects backward where it articulates laterally with the right maxilla. Of the alveoli, the second premaxillary alveolus, pm 2, is the smallest whereas pm 4 is the largest. Pm 1 appears to project nearly directly out of the front of the snout (procumbent) whereas the remaining alveoli appear to project ventrolaterally. There is no foramen for the lower dentary tooth present on the premaxilla. The left premaxilla was not recovered.

**Figure 5:**
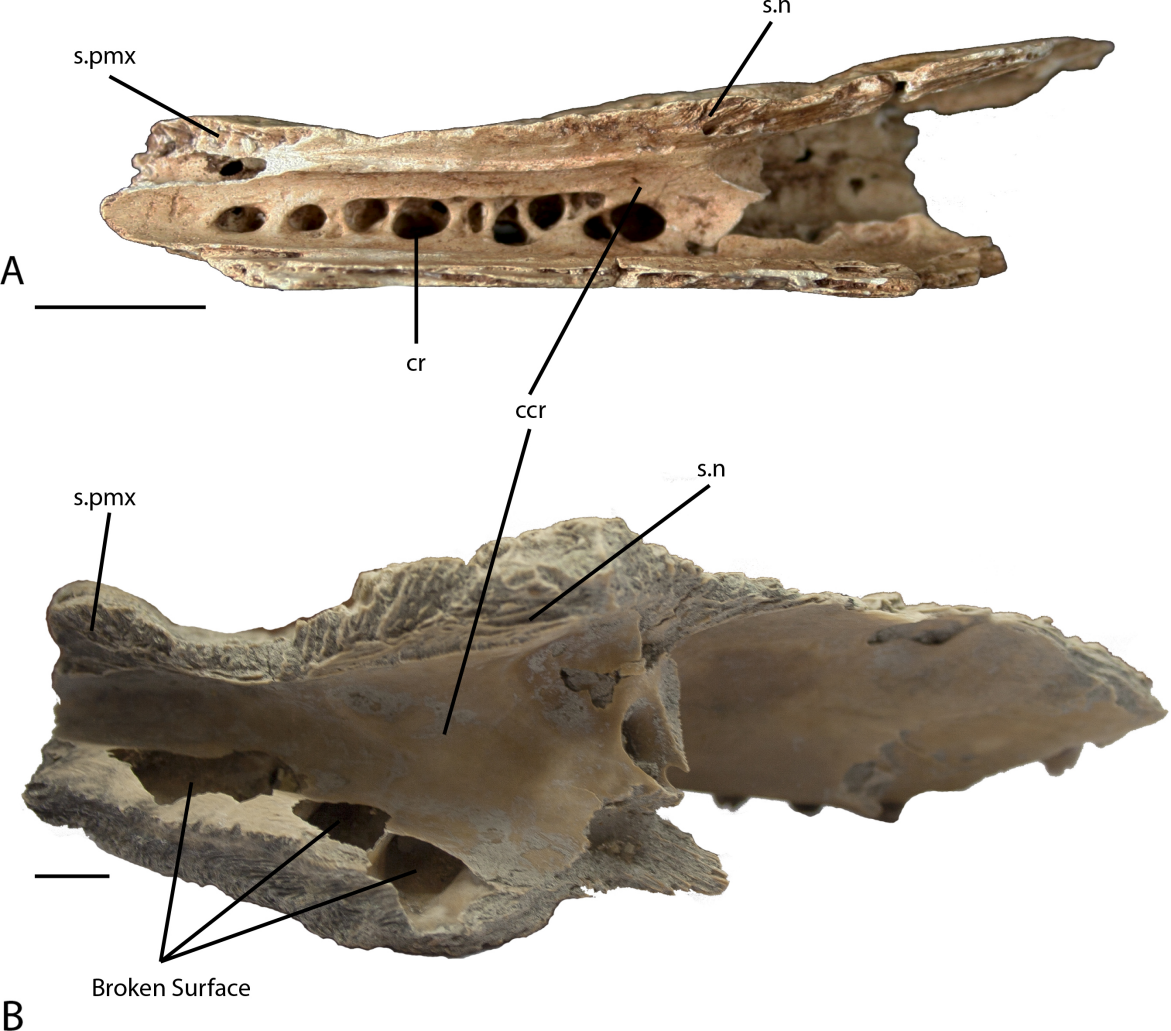
Right maxilla. Medial view of the right maxilla showing the wall of the caviconchal recess. (A) UAP-03.791 Anjohibe Cave specimen. (B) Uncatalogued UA specimen, *Voay robustus*. Abbreviations: ccr, medial wall of the caviconchal recess; cr, caecal recesses on the wall of the caviconchal recess; s.n, sutural surface with the nasal; s.pmx, sutural surface with the premaxilla. Scale = 1 cm.

The right maxilla is nearly complete and preserves 11 alveoli with what appears to be a broken 12th (Fig. 3C). The maxilla narrows at the maxillary tooth 4, m 4, alveolus where the notch for the dentary tooth 4, is present. The m 5 alveolus is the largest in the tooth row and is circular in shape. Posteriorly the alveoli become smaller and more oval in shape. One tooth remains in the m 9 alveolus. Occlusion pits are present between the alveoli for acceptance of the lower tooth row. As with most crocodylians, the dorsal surface bears a sculptured texture. Medially, the caviconchal wall of the maxilla is well preserved and bears a linear series of shallow recesses (Fig. 5).

The left maxilla is less complete, however it does preserve a nearly complete length, missing only its articular surface with the premaxilla and sutural contact with the ectopterygoid (Fig. 3C). The external wall of nearly all of the alveoli are broken and missing, however the presence of at least 10 alveoli can be confirmed. The alveolus for m 5 is the largest in the tooth row. Occlusion pits are not preserved. As with the right maxilla, a linear series of shallow recesses are present on the medial caviconchal wall.

The nasal bones are long and slightly convex laterally and contribute over two-thirds to the length of the snout (Fig. 3A). A V-shaped anterior process extends forward where they articulate with the premaxillae posterior to the external naris, widening posteriorly along their length, narrowing where they unite with the lacrimal, prefrontal and frontal. The nasals contact the maxillae along an underlapping suture along its length, and makes up the dorsal surface of the nasal passage.

Both left and right jugals are preserved and nearly complete (Fig. 3A). They form the lateral margin of the orbit and the ventral margin of the infratemporal fenestra. Anteriorly and anteroventrally they contact the maxilla along an overlapping suture. The anterior ramus of the jugal unites with the maxilla above the last four to five alveoli in the maxillary tooth row. The anterior ramus of the jugal further contacts the lacrimal dorsomedially. Medially, an ascending process rises from the center of the jugal forming the ventrolateral half of the postorbital bar. The postorbital bar forms the posterior margin of the orbit and the anterior margin of the infratemporal fenestra. The posterior ramus of the jugal forms the ventral border of the infratemporal fenestra and unites with the quadratojugal posterodorsally. The jugals are widest along their middle at the postorbital bar, and taper along their anterior and posterior rami.

Both lacrimals are preserved. The left lacrimal is missing roughly half of the anterior portion while the right is nearly complete (Fig. 3A). They are roughly half as wide as they are long and form the anterior margin of the orbit. They contact the jugal laterally, pre-frontals medially and the maxilla anteriorly. Along the margin of the orbit near the contact with the prefrontal, the lacrimal thickens. Along the anterior margin of the orbit, within the prefrontal-lacrimal suture lies the lacrimal duct foramen.

The prefrontals are triangular in shape with their length approximately twice their width (Fig. 3A). Medially the prefrontals contact the posterior process of the nasals and the anterior process of the frontal. Anterolaterally the prefrontals contact the lacrimals along a slightly concave suture due to a ridge at the orbit. They form the anteromedial margin of the orbit where they are thickened. The prefrontal pillars extend ventrally, and are relatively uniform in length. Midway along the length of the pillars a small process extends medially where it unites with the corresponding pillar forming an opening through which the olfactory tract runs.

The frontal bone sends an anterior process forward between the two prefrontal bones uniting with the posterior process of the nasals (Fig. 3A). The lateral margins of the frontal are thickened and form the medial border of the orbit. The sutural contact with the postorbitals and the parietal are sinuous and fairly distinct as compared with the other cranial sutures owing to its juvenile stage. The ventral surface bears a deep U-shaped trough running anteroposteriorly roofing the olfactory tract. Posteriorly the ridges of this trough terminate as two protuberances where they articulate with the laterosphenoids.

The postorbital is roughly kidney-shaped along its dorsal surface and contacts five cranial elements (Fig. 3A). A postorbital process descends ventrally where it contacts the ascending process of the jugal forming the dorsal half of the postorbital bar. The medial suture of the postorbital contacts the frontal along its anterior half and the parietal along its posterior half. The postorbital unites with the squamosal posteriorly on the lateral side of the main body along an overlapping suture. Ventrally it contacts the laterosphenoid immediately medial to the postorbital bar. The posterior margin of the postorbital forms the anterior border and one half of the lateral border of the supratemporal fenestra.

The parietal lies posterior to the frontal and forms the middle portion of the skull table, roofing the braincase (Fig. 3A). It is laterally expanded anteriorly where it unites with the frontal along its anterior margin and with the postorbitals laterally. The parietal constricts posteriorly forming an interfenestral bar that is flat dorsally and forms the medial walls of the supratemporal fenestrae. The dorsal surface of the interfenestral bar is narrow expanding ventrally, constricting the supratemporal fenestral opening. Posteriorly the parietal expands again articulating with the squamosals laterally and the supraoccipital on its posteroventral margin. A small V-shaped wedge of the supraoccipital is present on the dorsal surface of the skull table. Ventrally the parietal articulates with the laterosphenoids anteriorly and the quadrates posteriorly along the wall of the braincase.

The squamosal forms the posterolateral corner of the skull table and the posterolateral margin of the supratemporal fenestra (Figs. 3A and 3B). The posteriormost corners are upturned to form what appear to be incipient horns or “hornlets”. The structures are very small and seem to be covered with mineral deposits or possible preserved connective tissue, giving them the appearance of horns. The squamosal contacts the postorbital anteriorly along an underlapping suture that extends forward to the postorbital bar. Medially, the squamosal contacts the parietal behind the supratemporal fenestra and dorsal to the temporal canal. While it lies very close to the supraoccipital, the squamosal does not make contact with it, instead forming the dorsal margin of the posttemporal fenestra. The squamosal is expanded laterally and posterolaterally where it forms the dorsal roof and part of the posterior wall of the external otic aperture. Ventrally the squamosal articulates with the quadrate anteriorly below the squamosal/postorbital suture and posterolaterally a squamosal process unites with the ramus of the quadrate. The same squamosal process unites with the paroccipital process of the exoccipital posteroventrally. The lateral surface of the squamosal bears a groove that houses musculature for the earflap.

One nearly complete right quadratojugal is preserved and bridges the space between the jugal and the quadrate at the posterolateral corners of the skull (Fig. 3B). Anteriorly it articulates with the posterior ramus of the jugal. Posteriorly it articulates with the ramus of the quadrate where it forms half of the posterior margin of the infratemporal fenestra. A small process extends anteriorly into the infratemporal space, however this is broken. The quadratojugal extends posteriorly nearly the entire length of the ramus of the quadrate however it terminates just prior to the distal end and has no contribution to the mandibular condyle of the quadrate.

Two completely preserved quadrates were recovered from the specimen. The quadrate forms both the posterolateral wall of the brain case and the floor and partial posterior margin of the external otic aperture (Figs. 3A–3C) . Anterior to the otic aperture lies the preotic foramen. Anterior to the preotic foramen a thin lamina forms the posterodorsal half of the posterior margin of the infratemporal fenestra. Dorsally, the quadrate articulates with the laterosphenoids, squamosal, and the parietal forming one half of the ventral wall of the supratemporal fenestra. Ventrally the quadrate expands forming the posterior and ventral margin of the trigeminal foramen, however both quadrates are damaged in this region and only a small portion of the posterior margin of the trigeminal foramen can be seen on the right quadrate. Ventral to the trigeminal foramen the quadrate articulates with the pterygoid and the basisphenoid. The ramus of the quadrate extends posteriorly articulating with the quadratojugal laterally and with the paroccipital process of the exoccipital medially before forming the mandibular condyle that articulates with the mandible.

The palatines, though incomplete, are long and narrow extending posteriorly from their articular surface with the maxilla at the level of tooth position 7 to unite with the pterygoids at the end of the tooth row (Fig. 3C). They form the lateral walls and floor of the nasal cavity and the medial border of the suborbital fenestrae. Anteriorly they expand laterally where they contact the maxilla and posteriorly where they contact the pterygoids. The left palatine preserves a small dorsal process that articulates with the prefrontal pillar; the right process is broken.

Both ectopterygoids were recovered and are nearly complete, missing only a small portion of the anterior maxillary process and small portions of the descending rami (Fig. 3C). The anterior process of the ectopterygoid articulates laterally with the maxilla throughout most of its length, running medial to the posterior maxillary tooth row and forming the lateral margin of the suborbital fenestra. The right ectopterygoid extends to the border of the tenth and eleventh maxillary alveoli. Although it is incomplete, the sutural surface on the maxilla ends at the middle of m 10 indicating that only the very tip of the anterior process is missing. The anterior process of the left ectopterygoid extends to about the same distance and similarly is missing only the anterior-most tip. The ascending process of the ectopterygoid articulates with the jugal and forms the lateral half of the lower half of the postorbital bar. The descending ramus of the ectopterygoid articulates posteroventrally with the pterygoid buttress and the main body of the pterygoid along an underlapping suture. As with the anterior processes, the descending rami are also missing their distal tips.

The pterygoid is the ventral-most part of the basicranium and forms the roof of the nasopharyngeal duct dorsal to the palatines (Fig. 3C). The juvenile pterygoids are incomplete, preserving the main body and pterygoid buttress. The anterior process is broken and only represented by fragments; in complete specimens the posterior portion of the pterygoids completely enclose the duct behind the suborbital fenestrae before exiting at the internal choanae. The anterior process splits, forming a septum with separate chambers that are floored by the paired palatines. The anterior process extends to the anterior margin of the suborbital fenestra. Posteriorly the pterygoid expands laterally forming wings that are dorsoventrally thickened and form a buttress at the articular surface with the ectopterygoid. Near the midline, dorsal to the internal choana, a thin lamina extends dorsally on each side forming a V-shaped saddle where the basisphenoid sits. Two posterior pterygoid flanges are located immediately posterior to the internal choana and ventral to the median eustachian foramen. The ascending lamina forming the saddle for the basisphenoid articulates laterally with the quadrate.

Both laterosphenoids are partially preserved. The laterosphenoids enclose the anterior portion of the braincase and meet at the midline. Anteriorly they contact the main body of the frontal dorsally where they form the ventral opening for the olfactory tract. Ventral to the olfactory opening they form the opening for the optic nerve. They articulate posteroventrally with the basisphenoid before contacting the quadrate forming the anterior border of the trigeminal foramen. Dorsally they contact the postorbital and parietal.

The supraoccipital lies on the dorsal midline of the occipital region (Fig. 3A). It is a heart-shaped element wedged between the exoccipitals above the foramen magnum and posteroventral to the parietal (Fig. 3D). A small V-shaped wedge is present on the dorsal surface of the skull roof. The lateral surface forms a protuberance on each side that is an attachment site for epaxial muscles. These protuberances further form the ventral border of the posttemporal fenestra through which occipital veins pass.

The exoccipitals are well preserved; they form the lateral walls and roof of the foramen magnum (Fig. 3D). Laterally they expand forming the paroccipital processes that articulate with the posterior ramus of the quadrates. The exoccipitals come together at the midline forming a small condyle immediately dorsal to the foramen magnum. Dorsally they articulate with the supraoccipital. Several foramina can be seen lateral to the occipital condyle. A small foramen punctures the lateral wall of the foramen magnum through which runs cranial nerve XII. Immediately lateral to cranial nerve XII is a larger foramen housing the vagus nerve. Ventral to the vagus foramen is the lateral carotid foramen.

A partially preserved basisphenoid was recovered. The basisphenoid nests between the dorsal processes of the pterygoids and forms the anterolateral walls and anterior floor of the braincase. It articulates dorsally with the laterosphenoids and posteriorly with the quadrate. Anteriorly a cultriform process extends forward over the body of the pterygoids, however it is broken and missing in this specimen. The posterior surface is broken; the median eustachian foramen is preserved ventral to the basioccipital tubera.

The basioccipital is well preserved in the specimen (Fig. 3D). The most prominent feature is the occipital condyle, which articulates with the vertebral column and forms the floor of the foramen magnum. Laterally it articulates with the exoccipital. Ventrally it bears a prominent dorsoventral ridge immediately below the occipital condyle with shallow fossae on each side. The basioccipital articulates anteroventrally with the basisphenoid and sits snugly in a V-shaped saddle of the basisphenoid. Anteriorly the dorsal surface of the basioccipital forms the floor of the posterior portion of the braincase. On the articular surface with the basisphenoid a small channel forms the medial wall of the lateral eustachian opening. Ventral to the occipital condyle an indentation is present that forms the roof of the median eustachian opening. The lateral eustachian openings lie at approximately the same level of the median eustachian opening, as is typical in *Crocodylus* (Brochu, 2000).

### Mandible

**Figure 6:**
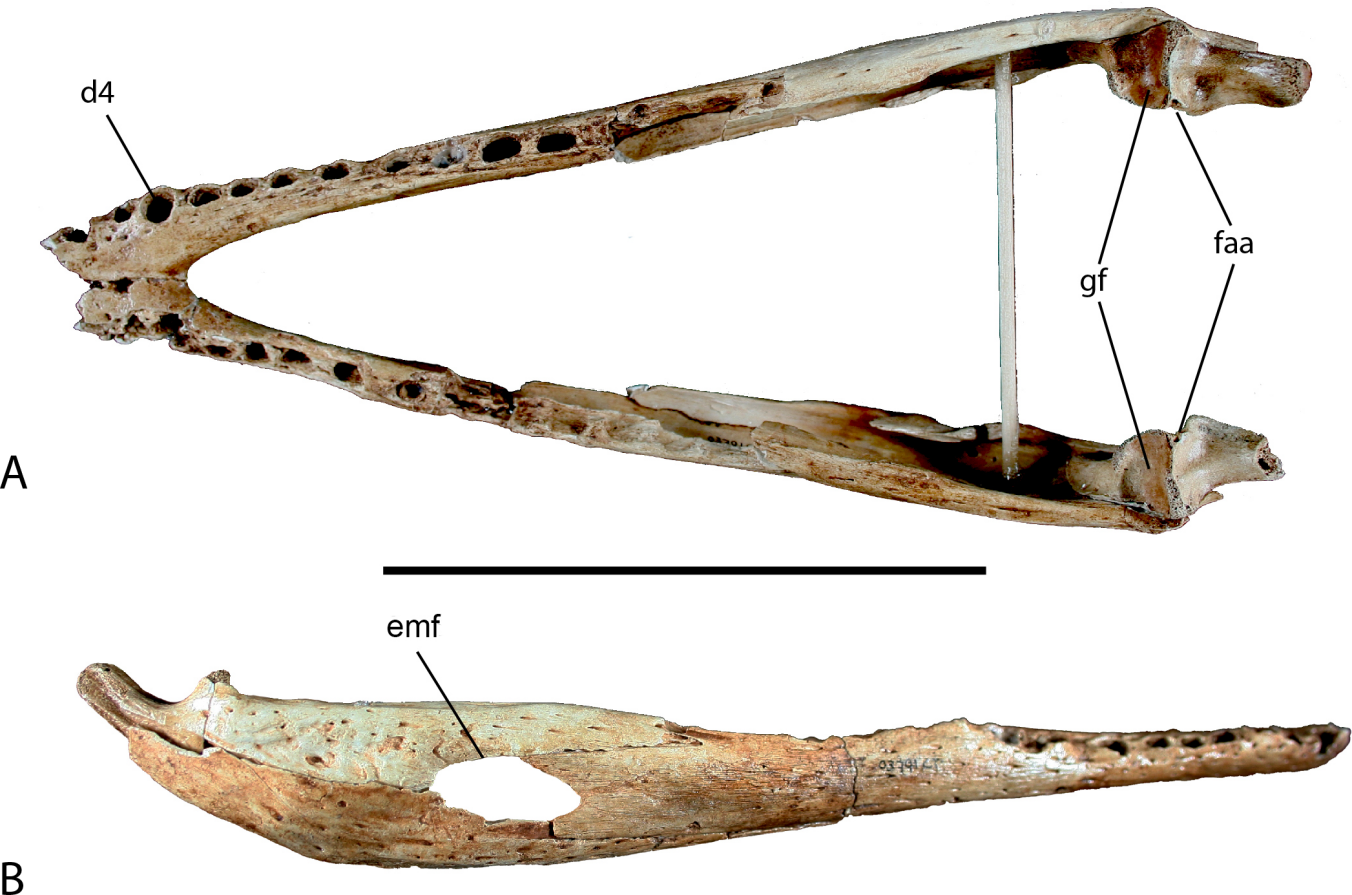
Mandible of UAP-03.791. (A) Doral view; (B) Right lateral view. Abbreviations: d4, alveolus for dentary tooth number 4; emf, external mandibular fenestra; faa, articular foramen aereum; gf, glenoid fossa of articular. Scale = 10 cm.

The dentary makes up the anterior two thirds of the length of the mandible (Fig. 6). Eleven complete alveoli are preserved in the right dentary with what appears to be three more in the posterior of the tooth row. The left dentary is less complete than the right preserving eight whole and four and a half partial alveoli. The anterior-most portion of the dentary is missing. However, because the fourth alveolus is the largest in the tooth row, and preserved on this specimen with two in front, only the anterior-most alveolus is missing on each side. The mandibular symphysis is nearly complete and extends posteriorly to the posterior border of the fourth alveolus. Posteriorly the dentary expands dorsoventrally where it contacts the surangular dorsally forming about one half of the dorsal margin of the external mandibular fenestra. Posteroventrally it articulates with the angular and contributes to one half of the ventral margin of the fenestra, however only a small portion is preserved. The Meckelian groove is preserved and runs along the medial dentary posterior to the symphysis expanding posteriorly.

Both splenials are incompletely preserved. The right is more complete, missing a small portion of the anterior and posterior ends. The left is fragmentary and represented only by a long sliver of the ventral margin and a smaller section of the dorsal margin. The splenial in crocodylids is a thin flat bone forming the medial wall of the mandible and Meckelian groove. It thickens on its posterodorsal margin where it forms the medial wall of the posterior tooth row. It articulates with the dentary laterally and the angular, surangular, and coronoid posteriorly, although only a small portion of the articular surface with the angular and surangular is preserved on both preserved elements.

The surangular forms the dorsal portion of the posterior end of the mandible and contributes one-third to the length (Fig. 6). Anteriorly it forms the posterodorsal margin of the external mandibular fenestra and contacts the dentary along an overlapping sutural contact where the dentary slots in medially. A medial process extends forward medially behind the dentary and bears an articular surface for the splenial. Posteriorly, the surangular articulates medially with the articular and forms the lateral wall of the glenoid fossa. Ventrally it contacts the angular behind the external mandibular fenestra along an underlapping suture.

The angular forms the ventral portion of the posterior end of the mandible and contributes about one half of the total length (Fig. 6). Anteriorly it unites with the dentary sending an elongate process ventral to the dentary and wrapping around to the medial side where it articulates with the splenial forming the floor of the Meckelian groove. Laterally it forms the ventral border and one half of the posterior border of the external mandibular fenestra. Posteriorly the angular ascends dorsally where it laterally overlaps the lower half of the articular and retroarticular process.

The articular is a small bone at the posterior-most end of the mandible and forms the articular joint with the skull (Fig. 6). The anterodorsal surface of the articular bears the glenoid fossa. The glenoid fossa is the articular surface for the quadrate. It is slightly constricted medially and flares laterally where it contacts the surangular. Two depressions are present on the surface of the glenoid where the mandibular condyles of the quadrate articulate and though they are of equal depth, the medial side is narrower than the lateral. Posterior to the glenoid fossa, the articular hooks posterodorsally with a concave surface forming the retroarticular process, which serves as the attachment for the *m. depressor mandibulae*. On the medial surface of the articular where the retroarticular process meets the glenoid is the foramen aereum. A small lingual foramen is located on the anterior surface of the articular ventral to the lateral fossa of the glenoid. Medial and ventral to the articular process is a deep trough for attachment of the *m. pterygoideus dorsalis*.

The coronoid is a small mediolaterally compressed bone of the lower jaw forming the posterior and ventral margin of the mandibular fossa and anterior margin of the medial intermandibular foramen. The left coronoid is complete whereas the right coronoid is missing a large portion of the anteroventral process as well as a small posteriorly directed process.

### Teeth

**Figure 7:**
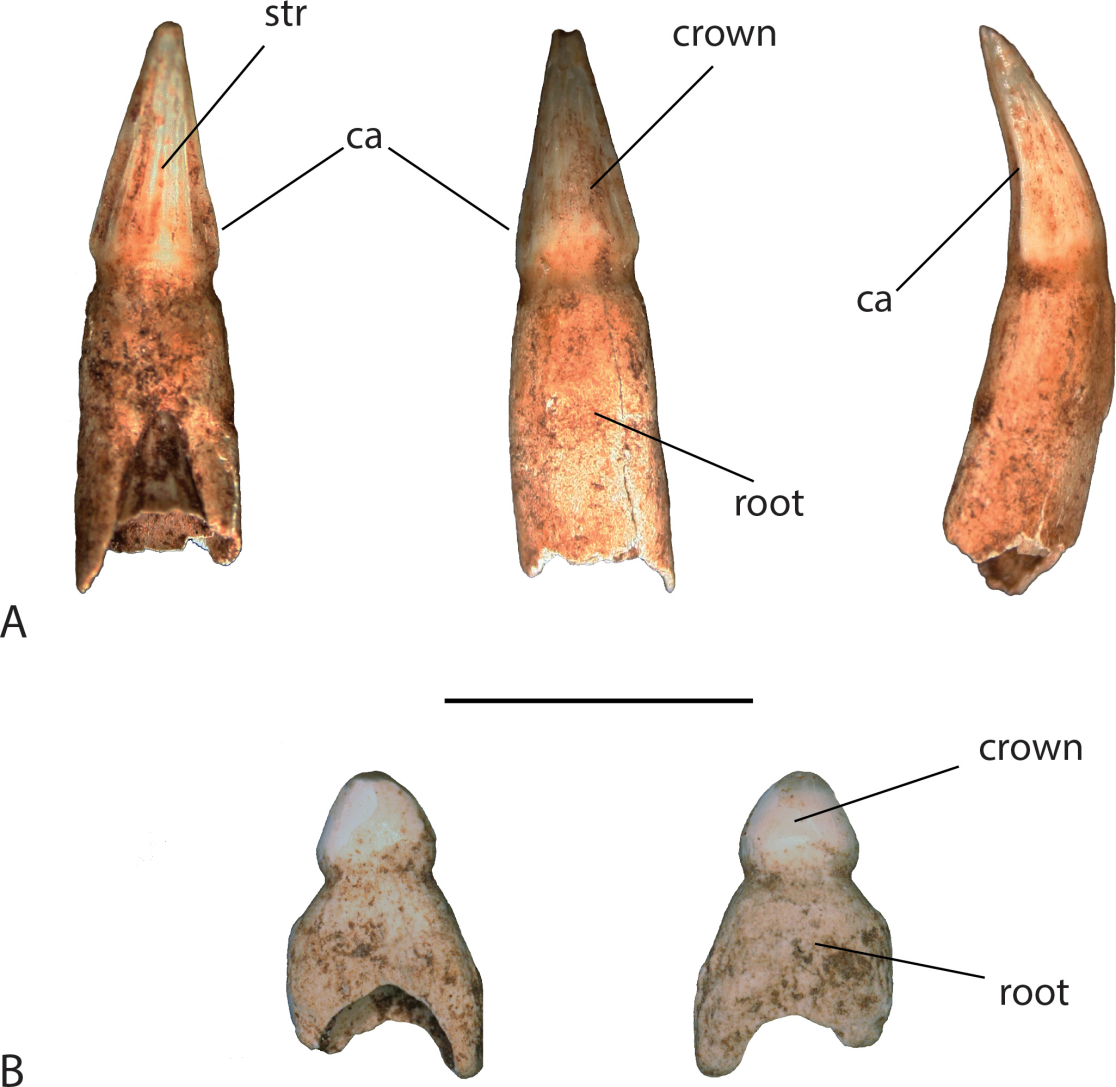
Teeth found in association with UAP-03.791. (A) anterior tooth in (l-r) lingual view, labial view, and lateral view; (B) posterior tooth in (l-r) lingual view and labial view. Abbreviations: ca, carinae; str, striations. Scale = 1 cm.

Only one tooth was found preserved *in situ* on the right maxilla. Thirty-eight teeth were found at the site in association with the remains. Nineteen of these had full or partially preserved roots, while the remaining 19 teeth were crowns only. Most of them are conical shaped with a weakly striated lingual surface and carinae on the anterior and posterior margin (Fig. 7). A few teeth from the posterior toothrow were recovered and are shorter and more bulbous than the conical anterior teeth (Fig. 7).

### Vertebral column

Most crocodylians have a vertebral formula of eight or nine cervical vertebrae, ten thoracic vertebrae, five lumbar vertebrae, two sacral vertebrae and about 30–40 caudal vertebrae (Griggs & Kirshner, 2015). The specimen described here preserves 23 complete or partial vertebrae (Fig. 2). Seven cervical vertebrae are preserved including a nearly complete atlas and a complete axis, 13 thoracic or lumbar vertebrae, one sacral vertebra, one sacral rib and one caudal vertebra. At least four anterior thoracic vertebrae can be positively identified by the presence of a keeled centrum and a hypapophysis on the anterior ventral surface. Mid to posterior thoracic vertebrae do not maintain these features and without the transverse processes cannot be distinguished from lumbar vertebrae. All of the recovered vertebrae are procoelous.

**Figure 8:**
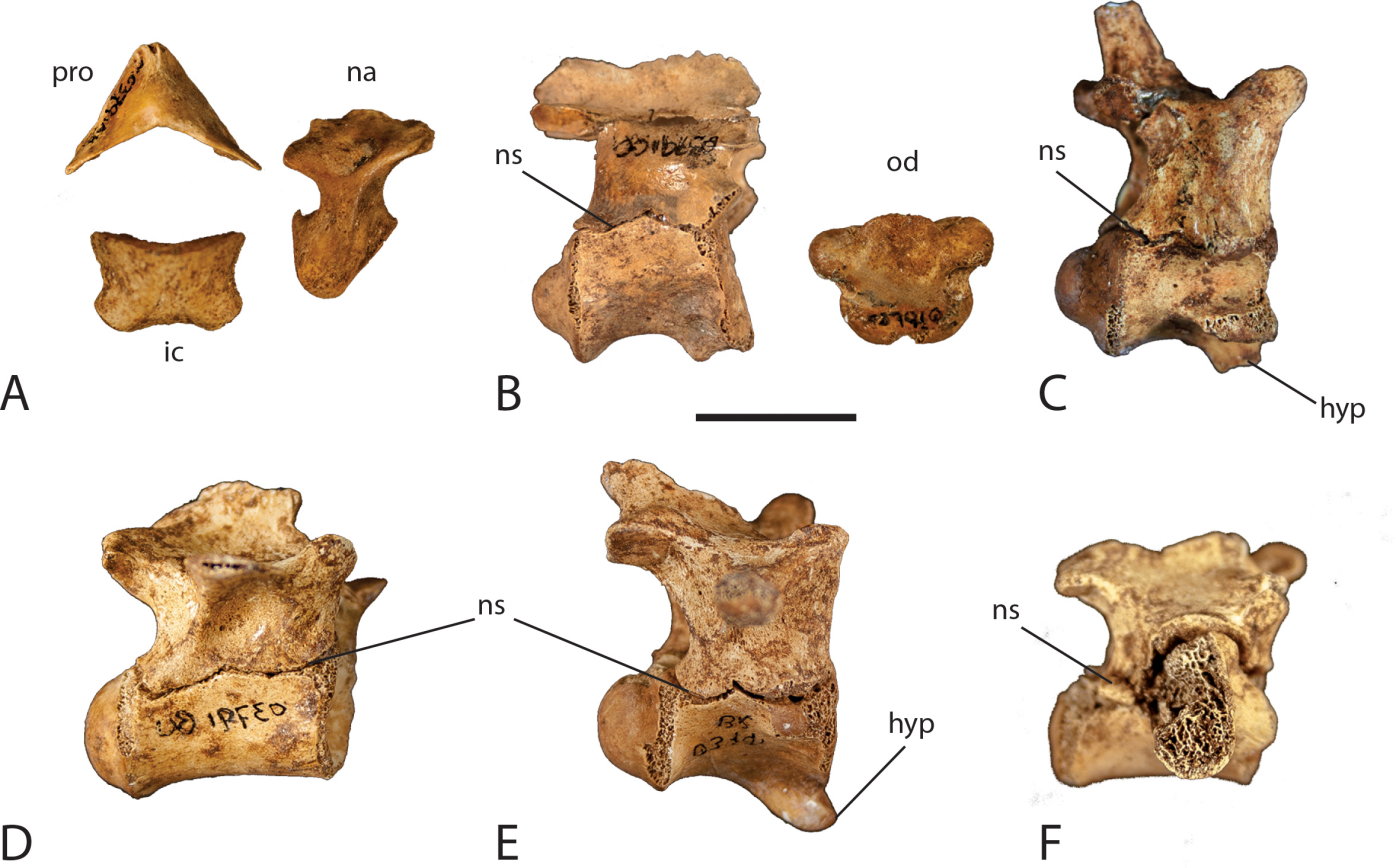
Neurocentral suture closure. Elements of the vertebral column showing degree of disarticulation and neurocentral suture closure. (A) atlas elements; (B) axis; (C) cervical vertebra 6; (D) posterior thoracic vertebra; (E) anterior thoracic vertebra; (F) first sacral vertebra. Abbreviations: hyp, hypapophysis; ic, intercentrum; na, neural arch; ns, neurocentral suture; od, odontoid process; pro, proatlas. Scale = 1 cm.

The atlas (C1) is not fused into one solid element like the rest of the vertebrae (Fig. 8A). It is made up of a dorsal proatlas, an intercentrum and paired neural arches (only the left lateral neural arch was recovered). The intercentrum is wedge-shaped with a concave dorsal surface and prominent diapophyses on the ventrolateral surface where the atlantal ribs articulate. The left neural arch rests above the intercentrum and forms the lateral wall of the neural canal. Ventrally it is robust with an articular facet on the anterior surface where it meets the occipital condyle of the skull. A second articular facet is present on the posterior surface where the arch contacts the odontoid process of the axis. The neural arch narrows dorsally before expanding anteroposteriorly and arching medially where it articulates with the proatlas on its anterior dorsal surface. The proatlas is a boomerang V-shaped bone forming the roof of the neural canal. It points anteriorly with the lateral “wings” terminating in articular facets where they contact the neural arches.

A complete axis (C2) with unfused odontoid process was recovered (Fig. 8B). The neural arch is attached to the centrum, however it is not completely fused; there is an open neurocentral suture. The centrum has a prominent ventral keel with a bifurcated hypapophysis anteriorly. The anterior sutural surface of the centrum is crenulated where it articulates with the corresponding surface of the odontoid process. Posteriorly the centrum forms a ball joint where it articulates with the adjacent vertebra. The neural arch is shallow dorsoventrally and possesses a short neural spine that runs the length of the vertebra. The prezygapophyses are small and weakly developed and positioned dorsolaterally where they articulate with the neural arch of the atlas. The postzygapophyses are larger and more developed, overhanging the neural canal at the posterior end of the neural spine. They are positioned ventrolaterally where they articulate with the prezygapophyses of the next vertebra. The odontoid process of the axis is disarticulated from the centrum. It is “petal” shaped with two articular facets on each side where the axial rib attaches. A short and stout process projects anteriorly from the anterodorsal surface.

There are five remaining cervical vertebrae preserved. Four are complete to nearly complete whereas one is a partial neural arch. They are similar in morphology to one another with minor differences. They are procoelous and have cylindrical shaped centra with a pronounced hypapophysis on the anterior part of the ventral surface (Fig. 8C). Lateral to the hypapophysis is a capitular facet that receives the capitulum of the cervical rib head. The neural arch is dorsoventrally expanded and bears a long and thin posteriorly projecting neural spine. Anterior to the neural spine, the paired prezygapophyses project forward with medially inclined articular surfaces. Posterior to the neural spine are the paired postzygapophyses that project posteriorly with ventrolateral articular surfaces. Along the anterior sutural surface with the centrum lies a small weakly developed ventrolaterally directed transverse process that receives the tuberculum of the cervical rib head. Proceeding posteriorly in the cervical series, the hypapophysis, capitular facets, transverse processes, and zygapophyses become more pronounced as the vertebrae increase in size and the ribs become larger.

Thirteen vertebrae representing the thoracic and lumbar region of the specimen are preserved. In crocodylians, thoracic and lumbar vertebrae are very similar in size and shape and are often indistinguishable from each other without the preserved transverse processes and diapophyses. The first four thoracic vertebrae can be identified by the presence of a well-developed hypapophysis on the anterior ventral surface of T1, decreasing in size to small and weakly developed on T4 (Fig. 8E). The remaining thoracic vertebrae lack a hypapophysis. As with the cervical series, all of the thoracic and lumbar vertebrae are procoelous with spindle shaped centra. T1 has a very pronounced hypapophysis that hooks forward. The capitular facet has migrated dorsally on the vertebra however it is still borne on the centrum at the level of the neurocentral suture. The presence of the capitular facet on each side gives the anterior condyle an oval shape. The neural arch is dorsoventrally expanded, however not to the extent seen in the cervical vertebrae. The neural spine is incomplete; however the base is sufficiently preserved to show that it is still quite thin. The prezygapophyses still maintain their dorsomedial articular surfaces although they are becoming more dorsally directed. Likewise, the postzygapophyses still maintain their ventrolateral articular surface; however they are becoming more ventrally directed. The transverse process is more developed than those in the cervical series and become more laterally projecting. They are not large however, and are rod-like in shape and located about half way up the neural arch.

Moving posteriorly in the vertebral column, the capitular facet continues to migrate dorsally spanning the neurocentral suture in T2 after which it is entirely born on the neural arch in the later vertebrae. The transverse process continues this pattern migrating dorsally where it begins near the top of the neural arch, just ventral to the neural spine in T2 and thereafter. The transverse process of T2 still maintains a rod-like shape, but the process is expanded anteroposteriorly in T4 and flattens out in the following vertebrae. As the capitular facet is now located on the neural arch, the centra become more circular in shape throughout the remainder of the vertebral column.

The remaining nine thoracic and lumbar vertebrae include two nearly complete vertebrae, three centra with partial neural arches, one complete centrum, one partial centrum, and two partial neural arches (Figs. 2 and 8D). None of them have preserved transverse processes so it is difficult to assign them to a specific region; based on size and shape, the majority seem to be from the thoracic region.

There are only two sacral vertebrae in crocodylians (Griggs & Kirshner, 2015). One nearly complete sacral vertebra (S1) and one partial sacral rib (S2) are preserved (Fig. 8F). Sacral vertebrae are different from the rest of the vertebral column in that they do not exhibit procoelous centra. The first sacral vertebra is concave anteriorly, as in all other vertebrae, however the posterior articular surface is flat. Likewise, the second sacral vertebra has a flat anterior articular surface and a concave posterior articular surface. The preserved specimen exhibits the concave anterior surface and flat posterior surface, which identifies it as S1. The centrum is wider than it is high and has an oval shape. The neural arch is relatively shallow dorsoventrally and lacks a preserved neural spine. The neural canal is smaller in size giving the neural arch a more heavily robust form. The prezygapophyses and postzygapophyses are well developed. The sacral ribs contact the vertebrae along a broad suture that spans half of the centrum and half of the neural arch. A partial right sacral rib is present on the specimen however the left sacral rib is missing. A partial sacral rib was recovered in the deposit that does not articulate with S1, suggesting it likely belongs to S2. The rib is too incomplete to determine which side of the vertebra it belongs.

One caudal vertebra has been identified from the site. It consists of a nearly complete centrum with a partial neural arch. The centrum is long and thin and spindle-shaped with typical procoelous form. The preserved neural arch is a thin lamina of the left lateral wall of the neural canal. A small broken surface of a transverse process is present on the neural arch. The shape, size, and presence of a transverse process suggest it is an anterior to mid-caudal vertebra. There is no visible sign of a neurocentral suture present on the vertebra, which is consistent with previous reports of juvenile crocodylians (Brochu, 1996; Ikejiri, 2012).

### Forelimb & pectoral girdle

The right scapula is incomplete, preserving most of the proximal end including the glenoid and articular surface with the coracoid and about one third of the scapular blade (Fig. 4C). The deltoid crest, while incomplete, is slender and located on the anterodorsal surface of the scapular body. The scapular blade narrows immediately behind the scapular body before expanding distally.

The left coracoid is nearly complete, missing only a small wedge from the distal blade and a small portion of the articular surface for the scapula (Fig. 4B). The coracoid is a flat bone with expanded proximal and distal ends. The body is pierced by the coracoid foramen anterior to the glenoid. The coracoid blade expands distally where it articulates with the sternum.

A partial left humerus was recovered from the site representing approximately two thirds of the proximal end (Fig. 4A). The humeral head is thin, but well developed. The deltopectoral crest is well developed though gracile.

Few elements from the manus were recovered. Those that were, include both the left and right radialae and one metacarpal (Figs. 4D–4F). The left radiale is complete whereas the right one is missing a small portion of the distal articular surface. The complete metacarpal is wider at its proximal end and has a slight twist distally.

### Hindlimb & pelvic girdle

The left ilium is fragmented and preserves the dorsal margin of the acetabulum and a portion of the posterior process (Fig. 4G). The supra-acetabular crest is thickened and rugose on its anterodorsal surface and becomes a small ridge as it passes posteroventally. The posterior process is incomplete and is thin and blade-like where preserved.

An element tentatively referred to as the left ischium preserves the proximal and mid-shaft to just prior to its distal expansion (Fig. 4H). Most of the acetabulum is missing with only a small portion of the middle section preserved.

The left pubis, while damaged, preserves most of its length missing only the distal end (Fig. 4I). Half of the proximal articular surface of the pubis is preserved. The pubic shaft narrows briefly before expanding toward the distal end, which is eroded.

What appears to be the left tibia is preserved as a nearly complete half (Fig. 4J). The epiphyses of the proximal and distal ends are not preserved, however the remaining length of the shaft is. Most of the entire one half of its length is missing or severely damaged. Due to the damage, this identification is tentative.

A possible left fibula was also recovered. It is nearly complete, missing only the distal articular surface (Fig. 4K). The proximal end is moderately expanded narrowing distally before expanding laterally at the distal end where it has been damaged. The proximal surface is semi-circular in cross-section. Although damaged, it appears that the distal surface exhibits the same semi-circular cross-section.

Some elements from the pes were recovered. Although exact identification of each element is difficult, three metatarsals, five pedal phalanges, and the left calcaneus were collected (Figs. 4L–4O). Two of the three metatarsals are complete and have been assigned to the right pes. The remaining metatarsal has been designated as from the left pes and is nearly complete, missing the distal condyle and a small section of the shaft. Four of the pedal phalanges are complete while one phalanx is nearly complete and missing only a small portion of the proximal articular surface. Three of the phalanges appear to be from the right pes, while the remaining two are from the left.

## Discussion

### Assessing maturity

Brochu (1996) documented that neurocentral suture closure in crocodylians progresses in a caudal to cranial direction. Most of the neurocentral sutures in the caudal vertebrae of *Alligator mississippiensis* Daudin, 1802, were closed or partially closed in all hatchlings whereas the neurocentral sutures in all presacral and sacral vertebrae were completely open. The odontoid process-axis centrum suture closes prior to the neurocentral suture in *A. mississippiensis* and *Crocodylus acutus* (Brochu, 1996). Additionally the sacral ribs remain separate from the vertebral body. This pattern of suture closure was consistent with other crocodylians including *C. acutus* Cuvier, 1807, *Osteolaemus tetraspis* Cope, 1861, and *Alligator sinensis* Fauvel, 1879 (Brochu, 1996). Using histological methods, Ikejiri (2012) further investigated neurocentral suture closure by examining the cartilaginous layers between the neural arch and the centrum, the synchondroses, through ontogeny in *A. mississippiensis*. This work corroborated the caudal to cranial pattern of neurocentral suture closure initially described by Brochu (1996). Interestingly, Ikejiri (2012) found that this pattern was reversed in the sacral vertebrae, with the anterior sacral fusing prior to the posterior sacral. While exploring allometric trends through ontogeny, Ikejiri (2015) also documented negative allometry in the diameter of the neural canal in *A. mississippiensis*. Hatchling specimens maintain a neural opening that is proportionally large relative to the centrum and vertebral height, whereas the neural canal in adult specimens is proportionally much smaller (Ikejiri, 2015). The specimen described here exhibits a complete lack of suture closure in all of the presacral and sacral vertebrae, most of which have completely detached neural arches (Fig. 8). The odontoid process is completely detached from the axis centrum. Only one caudal vertebra was recovered and there is no neurocentral suture visible. In all of the recovered vertebrae with complete or partial neural arches, the neural canal is large relative to the centrum and vertebral height. Although the neural canal is not as large as that seen in a hatchling, its size is consistent with what could be expected in a juvenile individual.

The degree of fusion or lack thereof exhibited by fossil skulls has often been used to characterize the growth stage of the animal. Numerous studies of non-avian dinosaurs have used fusion of the skull as a proxy for skeletal maturity (Sampson, Ryan & Tanke, 1997; Longrich & Field, 2012; Bailleul et al., 2016). While this has become a relatively common practice, Bailleul et al. (2016) found that this is not the case in crocodylians. Their study documented the sequence and timing of cranial fusion of skulls of the emu, *Dromaius novaehollandiae* Latham, 1790, and *A. mississippiensis* through ontogeny. While the pattern of cranial fusion in *D. novaehollandiae* follows an expected pattern of open sutures in young individuals becoming progressively closed as the animal matures, the inverse is found to occur in *A. mississippiensis*. As the alligator grows, the cranial sutures become wider, never allowing the elements to fuse together. While it is uncertain why this occurs, Bailleul et al. (2016) suggest that it may be attributed to species-specific biomechanics as the animal preys upon larger food sources. Although the skull of the described specimen was found in complete disarticulation and has been subsequently reassembled, it cannot be referred to a juvenile status based on this alone.

### Systematic placement

**Figure 9:**
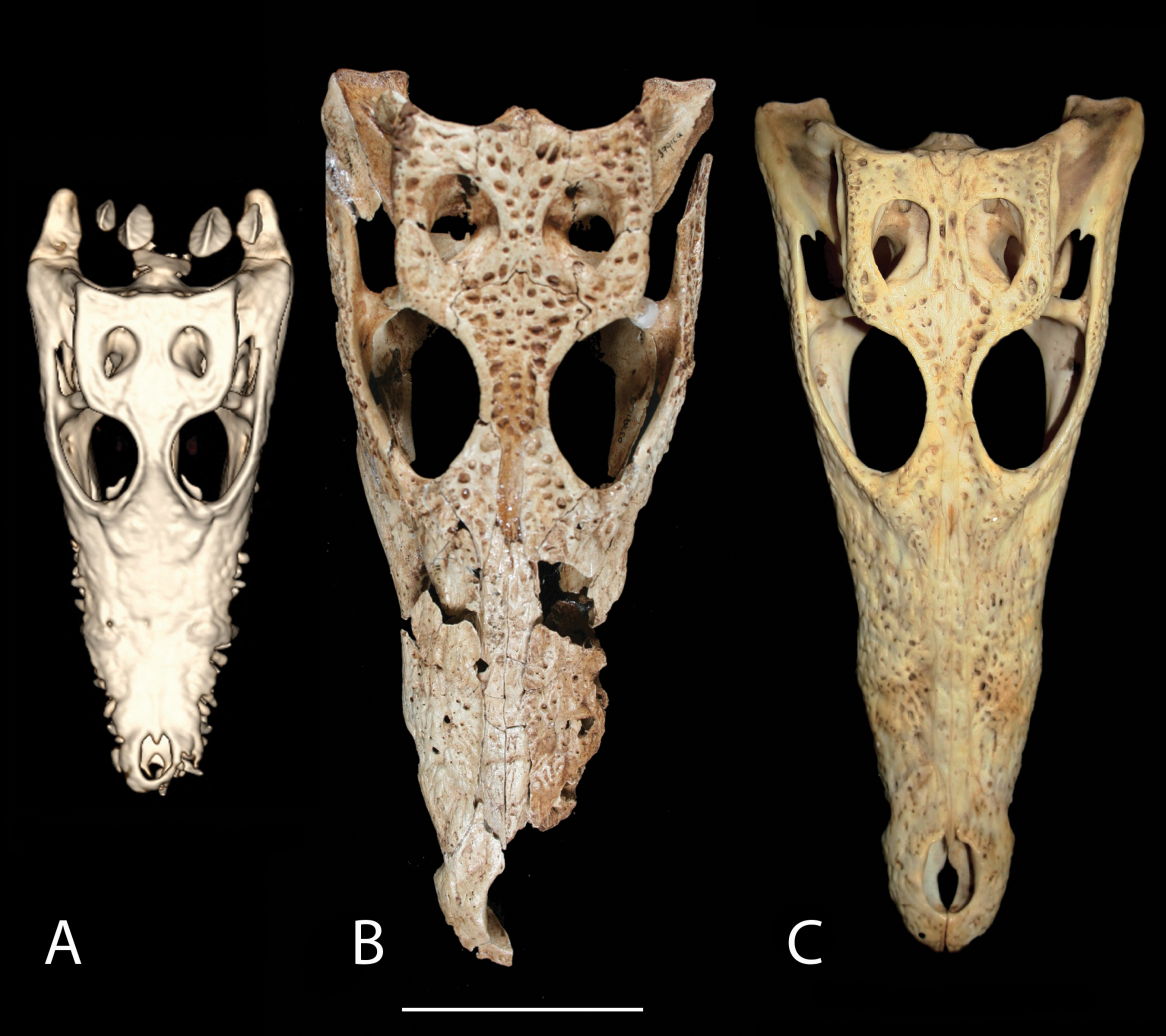
Skull shape comparison. Dorsal view. (A) FMNH 37216, *Crocodylus niloticus*. (B) UAP-03.791, Anjohibe Cave specimen. (C) UCMP 140796, *C. niloticus*. Scale = 5 cm.

The overall shape and morphology of the subfossil skull bears a strong resemblance to a juvenile *C. niloticus* (Fig. 9). UCMP 140795 and UCMP 140796 represent young *C. niloticus* ∼3.5 years of age. Other than a slight size difference these specimens appear to be nearly identical to the subfossil skull. It should be noted, however that snout shape is known to vary within crocodylian species and should not be used to make taxonomic judgment (Brochu, 2007). Additionally, because no known juvenile specimens of *V. robustus* are known, taxonomic placement based on shape alone should be avoided.

**Figure 10:**
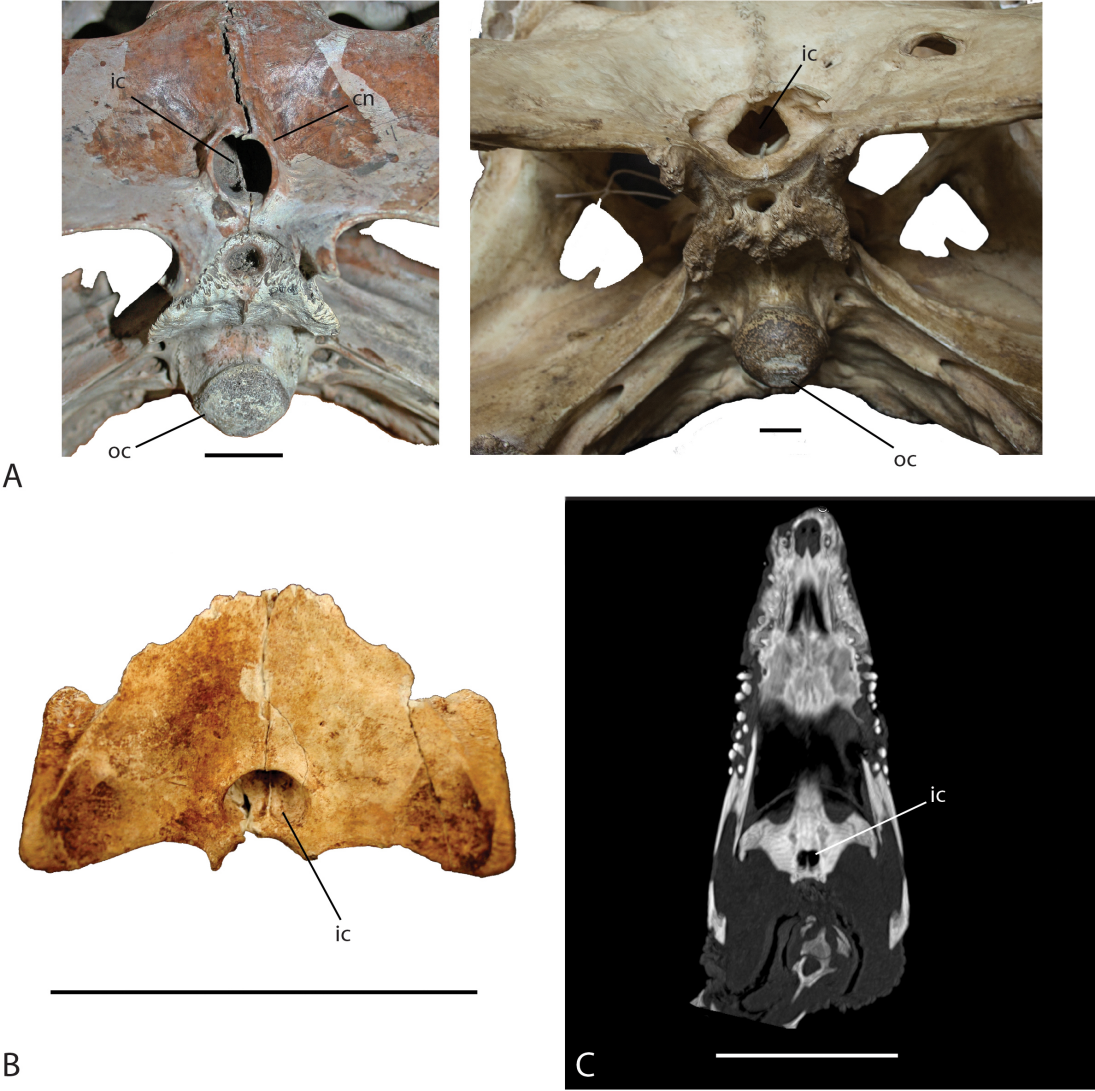
Ventral view of the internal choana of *Crocodylus niloticus* and *Voay robustus*. (A) uncatalogued UA specimen, *Voay robustus* (left) and uncatalogued UA specimen, *Crocodylus niloticus* (right), Scale = 2 cm. (B) Subfossil specimen UAP-03.791, Scale = 10 cm; (C) computed tomography image of *Crocodylus niloticus*, FMNH 37216, Scale = 5 cm. Abbreviations: cn, choanal neck; ic, internal choana; oc, occipital condyle.

The shape of the internal choana is a character used to distinguish between *C. niloticus* and *V. robustus* (Brochu, 2007). The internal choana of *V. robustus* has a distinct circular shape where it exits the pterygoids and has a pronounced choanal neck (Fig. 10). This characteristic is also seen in the extant species *Osteolaemus tetraspis* and *O. osborni* and is a distinct synapomorphy placing *Voay* within the Osteolaeminae (Brochu, 2007). The internal choana of *C. niloticus* on the other hand is oval in shape (Fig. 10) and lacks the distinct choanal neck that is seen in *V. robustus*. As illustrated in Fig. 10, the subfossil specimen exhibits the oval shape choana morphology suggesting it belongs to *C. niloticus*. Further evidence supporting this placement is FMNH 37216; a juvenile *C. niloticus* (Fig. 10) as well as those of UCMP 140795 and UCMP 140796. The internal choanae of these specimens are nearly identical to that of the subfossil specimen. It could be argued that this character could change through ontogeny in *Voay*, however Brochu (2007) noted that this condition is consistent throughout ontogeny in *Osteolaemus*. An examination of a growth series of skulls of *Crocodylus acutus*, with which *C. niloticus* share a close phylogenetic relationship, shows that the observed oval/D-shaped internal choana in this taxon does not change through ontogeny.

A second character that further supports the placement of the subfossil specimen within *C. niloticus* is the presence of well-developed caviconchal recesses on the medial wall of the maxilla. Both maxillae recovered in the subfossil specimen preserve a linear series of pockets projecting into the medial caviconchal wall (Fig. 5). In most crocodylians, the medial wall of the caviconchal recess is smooth, whereas extant *Crocodylus* possess the described pockets (Witmer, 1995; Brochu, 2000; Brochu, 2007). These recesses are not present in the maxilla of *V. robustus* and are a derived feature of the crown genus *Crocodylus* (Brochu, 2007). Because these cavities are absent from maxillae of *V. robustus* and *Osteolaemus*, and they are a derived feature of *Crocodylus*, it seems that this character lends unequivocal support toward referring the specimen to *C. niloticus*.

**Figure 11:**
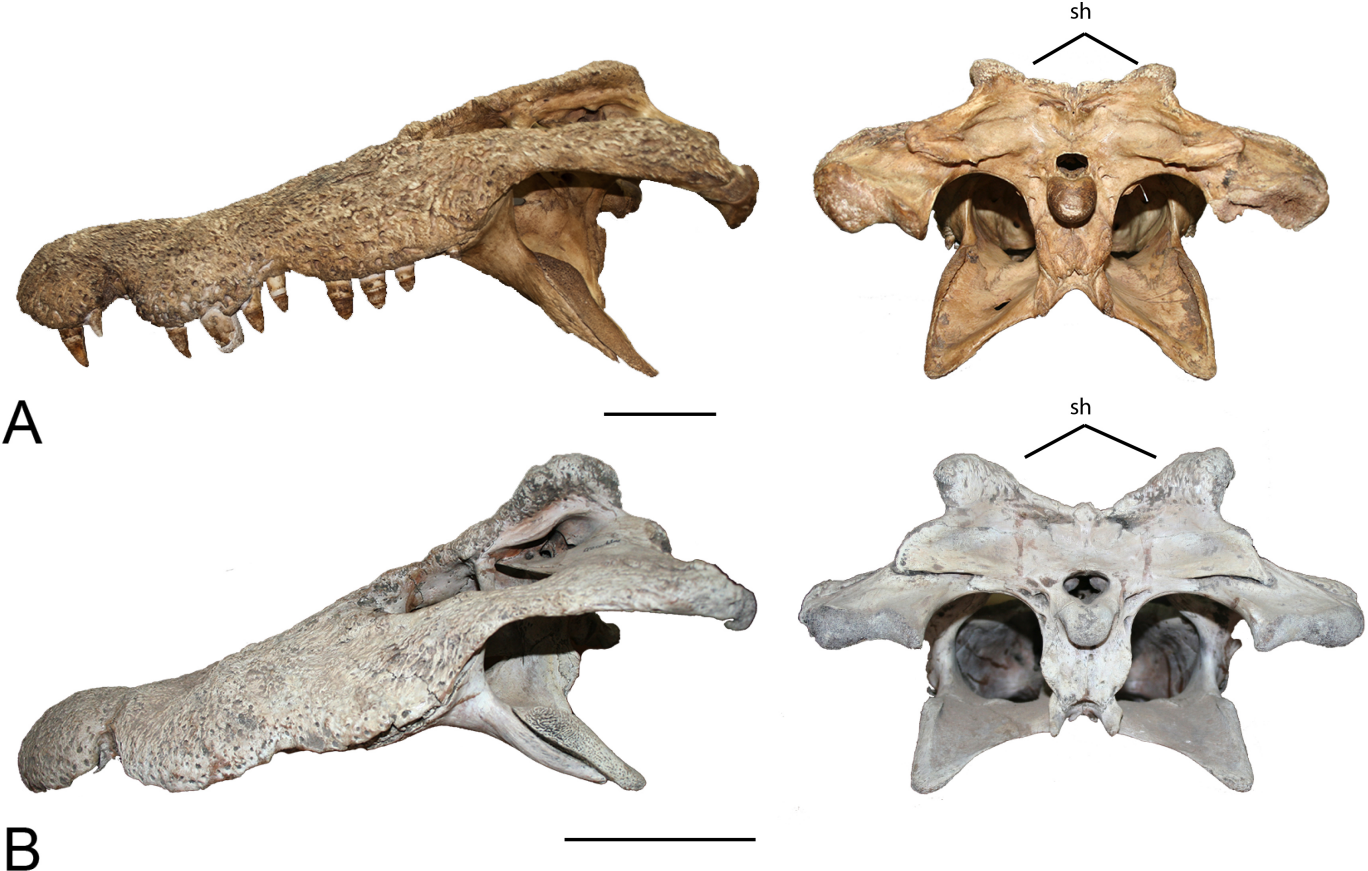
Squamosal horns. Left lateral view and posterior view of skulls showing degree of squamosal horn development. (A) uncatalogued UA specimen, *Crocodylus niloticus*. (B) uncatalogued UA specimen, *Voay robustus*. Skulls have been scaled to the same size for comparison. Abbreviation: sh, squamosal horn. Scale = 10 cm.

**Figure 12:**
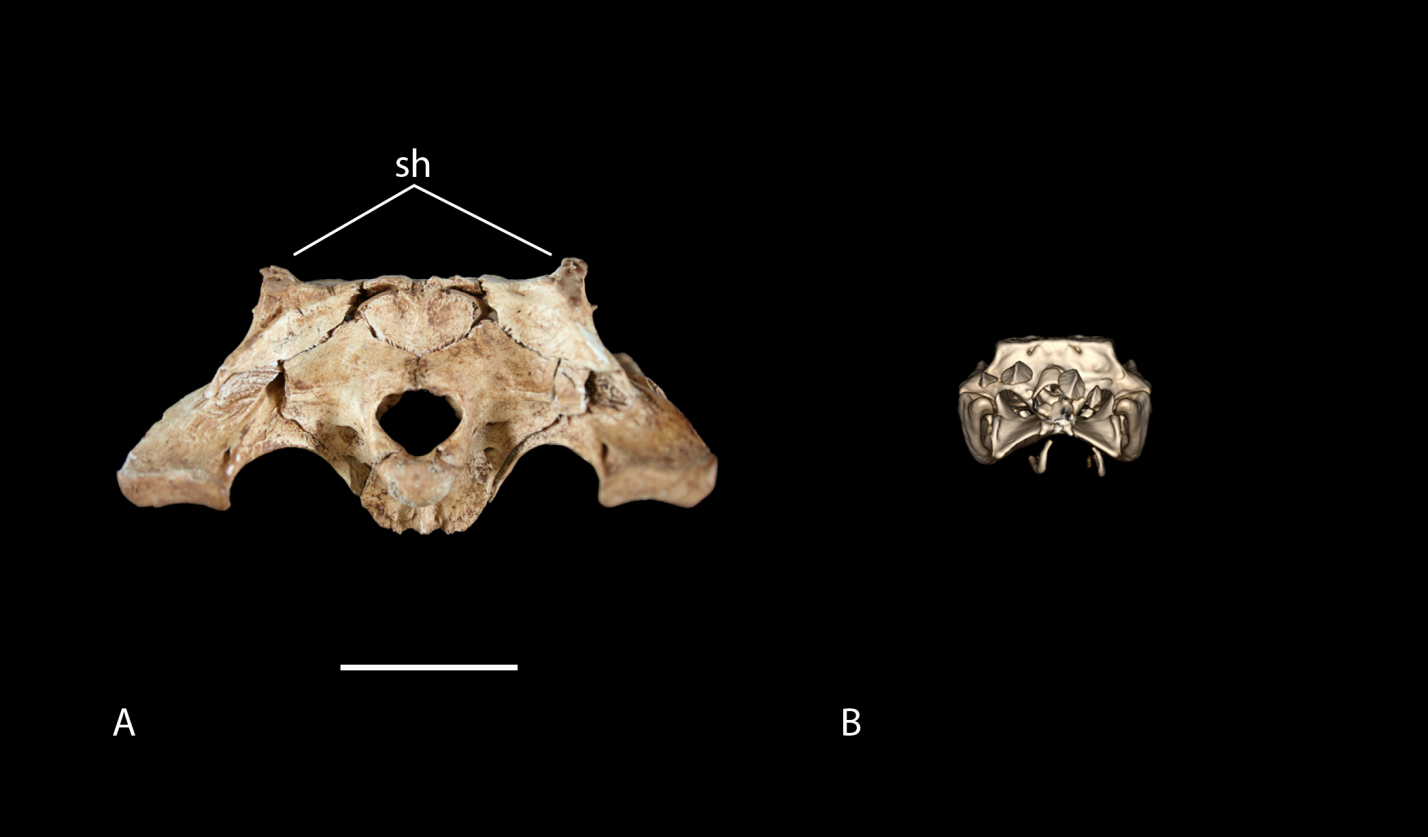
Posterior view of skulls showing degree of squamosal horn development in young individuals. (A) UAP-03.791, Anjohibe Cave specimen. (B) FMNH 37216, *Crocodylus niloticus*. Abbreviation: sh, squamosal horns. Scale = 5 cm.

One of the most prominent characteristics of *V. robustus* is the large squamosal horn on the posterolateral corner of the squamosal (Fig. 11). Squamosal horns are not uncommon among crocodylians and are present in extinct and extant taxa. In addition to *V. robustus*, extinct forms include *Crocodylus anthropophagus* Brochu et al., 2010, and *Aldabrachampsus dilophus* Brochu, 2006, while extant forms include *Crocodylus rhombifer* Cuvier, 1807, *Crocodylus siamensis* Schneider, 1801, and *C. niloticus* (Brochu, 2000; Brochu et al., 2010). Very old *Crocodylus porosus* have been documented having a very distinctly concave cranial table, suggesting the presence of squamosal horns (Mook, 1921a). Although squamosal horns can be observed in some adult *C. niloticus*, they are less prominent than they are in *Voay* and may be absent altogether. The Cuban crocodile, *C. rhombifer* and the Siamese crocodile, *C. siamensis* have demonstrated that squamosal horns vary through ontogeny (Delfino & De Vos, 2010). While mature adults are characterized by well-developed squamosal horns, juveniles’ horns are underdeveloped or absent (Brazaitis, 1973; Brochu et al., 2010; Delfino & De Vos, 2010). The subfossil specimen does appear to possess very small, yet distinct squamosal growths or “hornlets”. The horns appear to have a mineral deposit or preserved connective tissue on them which could give them a more pronounced appearance. While the CT image of the modern juvenile *C. niloticus* (FMNH 37216) lacks even the smallest amount of growth on the posterolateral corners of the squamosals, it should be noted that this specimen is very young and UCMP 140795, UCMP 140796 and UCMP 123091 all show squamosal “hornlets” similar to those seen in the subfossil specimen (Fig. 12). Because squamosal horns are not found exclusively in *V. robustus* and are known to show ontogentic variation (Brazaitis, 1973; Delfino & De Vos, 2010), their presence on the subfossil appear to be inconsequential.

The lack of discrete preorbital crests or rostral bosses distinguishes the specimen from inclusion within New World or Indopacific *Crocodylus* (Brochu, 2000; Brochu et al., 2010). In New World crocodiles such as *C. rhombifer* or *Crocodylus moreletii* A.H.A. Duméril & Bibron, 1851, elevated nasals impart a boss extending from the anterior lacrimals forward to the posterior premaxillae (Brochu, 2000). Likewise, Indopacific crocodiles, including *C. porosus* Schneider, 1802, and *C. siamensis* possess preorbital ridges on the dorsal surface of the snout (Mook, 1921b; Brochu, 2000). These features are not found on *C. niloticus* nor are they found on the preserved rostral bones of this specimen.

### Cryptic species

Until recently, there were thought to be only three species of crocodylians known throughout Africa and Madagascar (Schmitz et al., 2003): *Mecistops cataphractus* Cuvier, 1807, *Osteolaemus tetraspis*, and *Crocodylus niloticus*. The Nile crocodile, *C. niloticus*, was thought to be widespread across most of Africa and Madagascar (Schmitz et al., 2003). A surge of molecular studies over the last decade or so has aimed to elucidate relationships within Crocodylia in attempts to separate subspecies or to seek out potential cryptic species within established crocodylian populations (Ray et al., 2001; Schmitz et al., 2003; McAliley et al., 2006; Eaton et al., 2009; Hekkala et al., 2010; Hekkala et al., 2011; Meredith et al., 2011).

Using differing molecular techniques, it has been found that *C. niloticus* consists of two separate paraphyletic taxa that can be divided into East African and West African species (Hekkala et al., 2010; Hekkala et al., 2011; Meredith et al., 2011; Oaks, 2011). Recent analysis of Egyptian mummified crocodiles by Hekkala et al. (2011) found that they belong to the West African species, which supports previous arguments that the two species lived in sympatry along the lower Nile River. This provides strong support for referring the western clade back to the species *Crocodylus suchus* Geoffroy Saint-Hilaire, 1807, as originally described. Furthermore, Meredith et al. (2011) and Oaks (2011) found that the East African clade of *C. niloticus* shares closer affinities with New World crocodylians than it does with the western clade, providing strong evidence for trans-Atlantic dispersal of *Crocodylus* from Africa. Considering the distance required for a trans-Atlantic journey to reach the New World, the distance across the Mozambique Channel to Madagascar (∼450 km) seems much less daunting.

In the study by Hekkala et al. (2010), populations of *C. niloticus* exhibited genetic clustering that is consistent with Madagascar’s isolation from Africa. Interestingly, populations of *C. niloticus* sampled from northwestern Madagascar exhibit weaker divergence from those from the Zambezi River drainage in Zimbabwe than they do with other populations on the island (Hekkala et al., 2010). This could represent a more recent colonization, suggesting that *C. niloticus* dispersed to Madagascar through multiple dispersal events. Currently the timing of *C. niloticus* first reaching the island is poorly known; our subfossil specimen (460–310 ybp) represents the first dated subfossil crocodylian described from Madagascar, contributing a minimum date for arrival. Additionally, given its juvenile status, we infer that there must have been an established breeding population of *C. niloticus* on Madagascar by this date. As this specimen was discovered in the northwest of the island, further research using molecular methods could possibly determine if the specimen is consistent with results from Hekkala et al. (2010). Another possibility, however unlikely, is that the specimen could belong to *Crocodylus suchus*. Since it has been demonstrated that the two species lived alongside each other in the lower Nile River (Hekkala et al., 2011), there is the possibility that it could have dispersed to Madagascar as well. Although molecular data is needed to test this hypothesis, there is currently no evidence that *C. suchus* ever reached Madagascar.

## Conclusion

The subfossil crocodile specimen collected from Anjohibe Cave is a spectacularly preserved specimen and the only known juvenile crocodile represented in the subfossil record of Madagascar. While both *C. niloticus* and *V. robustus* have been documented from the cave (Goodman & Jungers, 2014), little effort has been made to systematically describe and identify the fossils. Although locally extinct from the cave, according to local residents *C. niloticus* was historically present in the cave (personal communication to KES). Although nothing is known about the growth and development of *V. robustus*, the skull of the subfossil specimen possesses characters that strongly support its placement within *Crocodylus*, and likely *C. niloticus*. Other than possible incipient squamosal horns that are evidently present in older specimens, the evidence is very convincing that it is indeed not a juvenile *Voay robustus*. Because the only remaining crocodylian known from the Cenozoic of Madagascar is *Crocodylus niloticus*, it seems parsimonious to consider this specimen a juvenile *C. niloticus* until further evidence suggests otherwise.

## Institutional Abbreviations

FMNH: Field Museum of Natural History, Chicago, Illinois, United States of America
UAP: Université d’ Antananarivo Paléontologie, Antananarivo, Madagascar
UCMP: University of California Museum of Paleontology—Berkeley, California, United States of America

## Additional Information and Declarations

### Data Availability

The following information was supplied regarding data availability: The research in this article did not generate any raw data.
